# Production of polyclonal antibody against the recombinant PirB^vp^ protein of *Vibrio parahaemolyticus*

**DOI:** 10.1186/s43141-021-00172-9

**Published:** 2021-05-11

**Authors:** Ngoc-Diem Duong, Khai-Hoan Nguyen-Phuoc, Kim-Yen Thi Do, Nguyet-Thu Thi Nguyen, Thuoc Linh Tran, Hieu Tran-Van

**Affiliations:** 1 University of Science, 227 Nguyen Van Cu Street, Ward 4, District 5, Ho Chi Minh City, Vietnam; 2 Vietnam National University, Ho Chi Minh City, Vietnam; 3 Pasteur Institute, 167 Pasteur Street, Vo Thi Sau Ward, District 3, Ho Chi Minh City, Vietnam

**Keywords:** AHPND, Polyclonal antibodies, ELISA, *Vibrio parahaemolyticus*, PirB^vp^ protein, Recombinant protein

## Abstract

**Background:**

Acute hepatopancreatic necrosis disease (AHPND) is caused by toxin-producing strains of *Vibrio parahaemolyticus* which contain deadly binary toxins PirA^vp^ and PirB^vp^ encoded in pVA1 plasmid. The polyclonal antibodies against PirB^vp^ protein could be used to develop immunochromatographic test strip for in-field diagnosis of AHPND.

**Results:**

In this study, PirB^vp^ gene was amplified, cloned, and expressed in *E. coli*. The expressed protein was detected by sodium dodecyl sulfate-polyacrylamide gel electrophoresis (SDS-PAGE) and Western blot probed with 6xHis antibodies. Then, the recombinant PirB^vp^ (rPirB^vp^) was purified using Ni-Sepharose column. Rabbits were immunized with the purified rPirB^vp^, and produced antibodies were analyzed using Ouchterlony double immunodiffusion. The antibody titration and antibody purification were performed by ELISA and affinity chromatography, respectively. Finally, antibody specificity and sensitivity were evaluated by dot blotting. The present study showed a high titer of polyclonal antibodies in rabbit serum after immunization and the titer increased steadily during the immunization schedule. The highest titer of antibody reached up to 2,560,000 with LOD of 0.1 ng/mL. The purified antibodies showed no cross-reactivity with proteins from other *Vibrio* species, and the detection threshold ranged from 6.25 to 12.5 ng toxin/dot.

**Conclusion:**

This study highlights the production of high titer and specific polyclonal antibodies as an initial material towards the development of lateral-flow strip test.

## Background

Acute hepatopancreatic necrosis disease (AHPND) is a serious disease that has caused severe damage and significant financial losses to the global shrimp industry [1, 2]. Since its first appearance in China in 2009, AHPND has quickly spread to Vietnam in 2010, Malaysia in 2011, Thailand in 2012, Mexico in 2013, the Philippines in 2015, and South America in 2016 [3]. AHPND can cause up to 100% mortality within approximately 35 days after stocking shrimp post-larva in farmed ponds. Typical symptoms of affected shrimp include an empty gut, and atrophied hepatopancreas. Histopathological analysis shows sloughing of the hepatopancreatic tubule epithelial cells and hemocytic infiltration [4].

In 2013, the causative agent of AHPND was identified as unique strains of *Vibrio parahaemolyticus* (VP_AHPND_) that carry a 70-kb plasmid encoding for binary toxins PirA^vp^/PirB^vp^, which are homologous to the *Photorhabdus* insect-related toxin [5, 6]. PirA^vp^/PirB^vp^ are secreted toxins that were determined to be the primary virulence factors causing AHPND. PirB^vp^ (a 50.1-kDa protein) alone is capable of inducing AHPND histopathology in hepatopancreatic tubules, while PirA^vp^ (a 12.7-kDa protein) causes only minor histological changes [7].

The current molecular methods for detection of VP_AHPND_ isolates are mostly based on PCR which target the PirA^vp^/PirB^vp^ toxin genes, but it is difficult to apply these methods in farmed ponds *in situ* [7, 8]. Therefore, antibody-based methods have been deployed for the pathogen detection, e.g., Western blot and ELISA methods are used for detection of PirA^vp^/PirB^vp^ toxin based on monoclonal antibody [9]. However, production of monoclonal antibody is significantly more expensive, time-consuming, and requires additional maintenance of cell lines. Therefore, this study was conducted for production, purification, and evaluation of the rabbit polyclonal antibodies against recombinant PirB^vp^ protein (rPirB^vp^). The produced polyclonal antibodies were also assessed for their sensitivity and specificity to rPirB^vp^, native counterpart as well as other *Vibrio* species. This work provides the prelude for immunochromatographic test strip development for AHPND diagnosis.

## Methods

### Materials

AHPND strain of *Vibrio parahaemolyticus* XN89 and non-AHPND strain XN8 were kindly provided by Dr. Saengchan Senapin National Center for Genetic Engineering and Biotechnology (BIOTEC), Thailand. *Vibrio cholerae*, *Vibrio vulnificus*, *Vibrio alginolyticus*, and White Spot Syndrome virus were kindly provided by Dr. Tuan V. Vo from University of Agriculture and Forestry, Ho Chi Minh City, Vietnam. Female New Zealand white rabbits were provided by Central Animal House of Pasteur Institute in Ho Chi Minh City. PCR kit was product of Bioline, USA. Restriction endonucleases, DNA, and protein markers were all purchased from Thermo Scientific, USA. Ni-sepharose and Hitrap protein G HP column were purchased from Cytiva Life Sciences, Sweden. Culture media were purchased from BD. All other used chemicals were of analytical grade, unless otherwise specified.

### Bacterial strains and growth conditions

All *Vibrio* strains were grown routinely in tryptic soy broth (BD Difco) containing 1.5% NaCl at 30 °C.

### Preparation of recombinant PirB^vp^ protein

#### Amplification and cloning of PirB^vp^

Plasmid DNA extracted from *V. parahaemolyticus* XN89 by using commercial plasmid isolation kit. Due to *Nde*I restriction site located within PirB^vp^ gene, recombinase-free cloning in which designed primers with 15-bp overlap homology at their ends to cloning vector was exploited. The designed primers used for the amplification of PirB^vp^ gene included ToxB-F (5’-taagaaggagatataCATATGACTAACGAATACGTTGTAAC-3’) and ToxB-R (5’-gtggtggtggtggtgCTCGAGCTTTTCTGTACCAAATTCATC-3’). The resulting amplicon was incubated with *Nde*I-*Xho*I-treated pET22b vector at RT for 30 min, then transformed into chemically competent *Escherichia coli* DH5α cells. The recombinant plasmid was confirmed for accurate insertion by both restriction enzyme digestion and sequencing.

#### Expression of recombinant PirB^vp^ protein

The pET22b-PirB^vp^ plasmid was transformed into *E. coli* BL21 (DE3) [10]. Colonies of *E. coli* BL21 (DE3) containing the pET22b-PirB^vp^ plasmid was inoculated into 10-mL Luria broth media containing 50 *μ*g/mL ampicillin and incubated overnight at 37 °C with shaking at 200 rpm. The expression of recombinant protein (rPirB^vp^) was induced by 0.5 mM IPTG and harvested by centrifugation. A volume of 1.5 mL bacterial broth was pelleted, mixed with 300 μL PBS, sonicated, and centrifuged to collect total protein, soluble protein, and insoluble protein fractions. Expression of the recombinant protein was confirmed by SDS-PAGE with Coomassie blue staining and Western blotting probed with anti-6xHis tag. Control samples were total fraction of *E. coli* BL21 (DE3)/pET22b with IPTG induction and *E. coli* BL21(DE3)/pET22b-PirB^vp^ non-IPTG induction. After that, *E. coli* BL21 (DE3)/pET22b-PirB^vp^ strain was expressed and scaled up to 100 mL LB medium for collecting rPirB^vp^. Soluble protein fraction was purified by affinity chromatography method with Histrap HP column (Cytiva Life Sciences). The column was loaded with 20 mL total soluble fraction. Next, the column was washed with binding buffer (20 mM phosphate 0.5M NaCl, 40 mM imidazole, pH 7.4). Finally, the target protein was eluted with elution buffer (20 mM phosphate, 0.5M NaCl, 108 mM imidazole, pH 7.4). The result of purification was verified by SDS-PAGE with silver staining and analyzed by a gel analyzer software. The concentration of obtained protein was determined by the Bradford method.

### Immunization of rabbits and production of polyclonal antibodies

Healthy, 12-week-old, female New Zealand rabbits were maintained in the experimental animal facility, and experiments were performed in accordance with the Directive 2010/63/EU guideline approved by The Animal Care and Use Committee of University of Science, VNU-HCM in Ho Chi Minh City (ethical code 12/18-0599-01). The animals were housed singly in suspended cages and fed chow and water ad libitum. Three rabbits were injected subcutaneously with an emulsion of 1 mL rPirB^vp^ in PBS, and 1 mL of Complete Freund’s Adjuvant at the first dose. Four booster injections of the same protein mixed with Incomplete Freund’s Adjuvant were given to each rabbit on a monthly basis. The rabbits were bled via the marginal ear vein prior to the first dose and two-week intervals, and serum was tested to detect antibodies against rPirB^vp^. Rabbits after completion of the experiment were anesthetized subcutaneously with Xylazine (5 mg/kg) for 5 min then Ketamine (40 mg/kg) followed by exsanguination to euthanize. After experiments, the rabbits were disposed and cremated following local regulations.

### Detection of antibody by the Ouchterlony double immunodiffusion technique

Antibodies were detected using double immunodiffusion [11]. Petri plates containing casted agarose with six peripheral wells contained serially diluted serum, and a central well contained recombinant and native PirB^vp^ antigen. Antigen and serum were filled into wells (20 μL per well). The double diffusion plates were stored in a humidified chamber at 4 °C, and the precipitin lines were examined daily for 2 days.

### Detection of antibody by ELISA

Immunized rabbit sera after four injections were detected by coating the recombinant and native PirB^vp^ proteins at 0.5 μg/mL in 0.1 M carbonate buffer, pH 9.6 overnight at 4 °C into 96-well ELISA plates. The plates were washed four times with PBS-0.01% Tween 20 (PBS-T) and blocked with 1% bovine serum albumin in PBS for 30 min at 37 °C. After a washing step, 100 μL of 1:1000 diluted serum was added to triplicate wells, and the wells were incubated for 1 h at 37 °C. Unbound antibody was removed by washing four times with PBS-T, and 100 μL of 1:40,000 diluted goat anti-rabbit IgG peroxidase conjugate (Sigma-Aldrich) was added to each well and incubated for 30 min at 37 °C. After washing four times with PBS-T, the wells were reacted with 3,3′,5,5′-Tetramemethylbenzidine (TMB) at room temperature in dark room for 10 min. The reaction was stopped by 50 μL 1N H_2_SO_4_. Absorbance was measured at 450 nm with a microplate reader.

### Purification and determination of affinity-purified antibodies

Immunized rabbit sera were collected and precipitated by 50% ammonium sulphate. After dialysis against PBS, immunoglobulins were purified by Hitrap protein G HP column (Cytiva Life Sciences). Immunoglobulins were applied through affinity column. Next, the column was washed with binding buffer until the absorbance reached the baseline. Then, the respective bound antibodies were then eluted with 0.1 M glycine (pH 3.0) into 0.1 M Tris (pH 11.0), thus minimizing exposure of the antibody to acid. The effluent was dialyzed against PBS, determined content by Bradford assay and analysed by SDS-PAGE. Finally, affinity-purified antibody titer was determined by the ELISA method as described above. The titer of each antibody sample was arbitrarily designated as the maximum dilution that yielded at least twice the absorbance of the same dilution of nonimmune control antibody [12].

## Characterization of affinity-purified antibodies

### Specificity of antibodies

Specificity of the prepared antibodies was evaluated by dot blotting. Supernatants from strains VP_AHPND_, VP_non-AHPND_, purified recombinant PirB^vp^ protein as well as other *Vibrio* species (*V. cholerae, V. vulnificus, V. alginolyticus*), White Spot Syndrome virus, and bovine serum albumin were fixed onto nitrocellulose membrane. This membrane was blocked with 3% skim milk. Next, the affinity-purified antibody (diluted 1:10,000) was added and incubated for 1 h. After washing, the membrane was incubated with HRP conjugated goat anti-rabbit IgG for 1 h. Finally, the membrane was washed as before and incubated for 15 min in TMB substrate.

### Sensitivity of antibodies

Sensitivity of the prepared antibodies was evaluated by dot blotting and ELISA. Proteins from VP_AHPND_, VP_non-AHPND_, and purified recombinant PirB^vp^ protein at initial concentrations of 500 ng/ml were serially two-fold diluted from 200 ng/mL to 3125 ng/mL and fixed onto nitrocellulose membrane. Then, the dot blot was performed as described in the previous section. For ELISA, the recombinant PirB^vp^ protein was diluted and coated onto 96-well ELISA plates at 0.0001–100 ng/mL (100 μL). Then, ELISA was performed as described in the previous section.

## Results

### Preparation of recombinant PirB^vp^ protein

#### Amplification and cloning of PirB^vp^

**Fig. 1:**
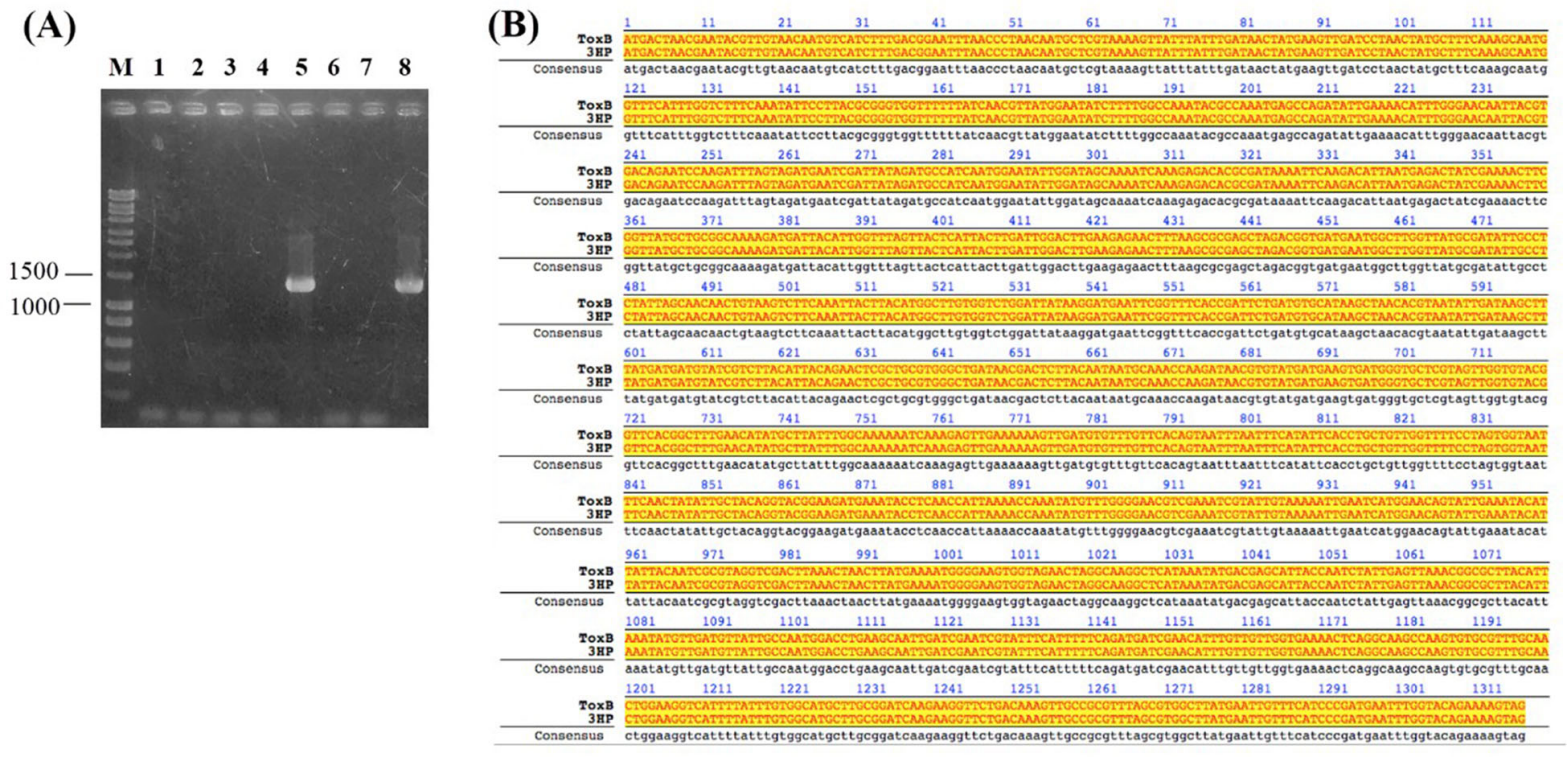
Molecular cloning of pET22b plasmid containing PirB^vp^ gene (**a**) and sequencing verification (**b**). **a** Lane M: marker; Lane 1: negative control; Lane 2–8: recombinant plasmid-containing candidate colonies. **b** Result of sequencing and homologous alignment between a typical positive clone with 3HP strain (GI: 1848399002)

Polymerase chain reaction was performed using PirB^vp^ gene-specific primers for *V. parahaemolyticus*. The expected size of amplified PirB^vp^ gene from AHPND-positive *V. parahaemolyticus* strains was approximately 1320 bp. After homologous recombination, candidate colonies were screened for recombinant plasmid. No amplification was observed from negative control (Fig. 1a). The result was confirmed again by sequencing plasmids obtained from positive colonies. Sequencing results showed that a typical recombinant clone obtained was 100% homologous to PirB gene (GenBank KP324996.1) (Fig. 1b).

#### Expression of recombinant PirB^vp^ protein

**Fig. 2:**
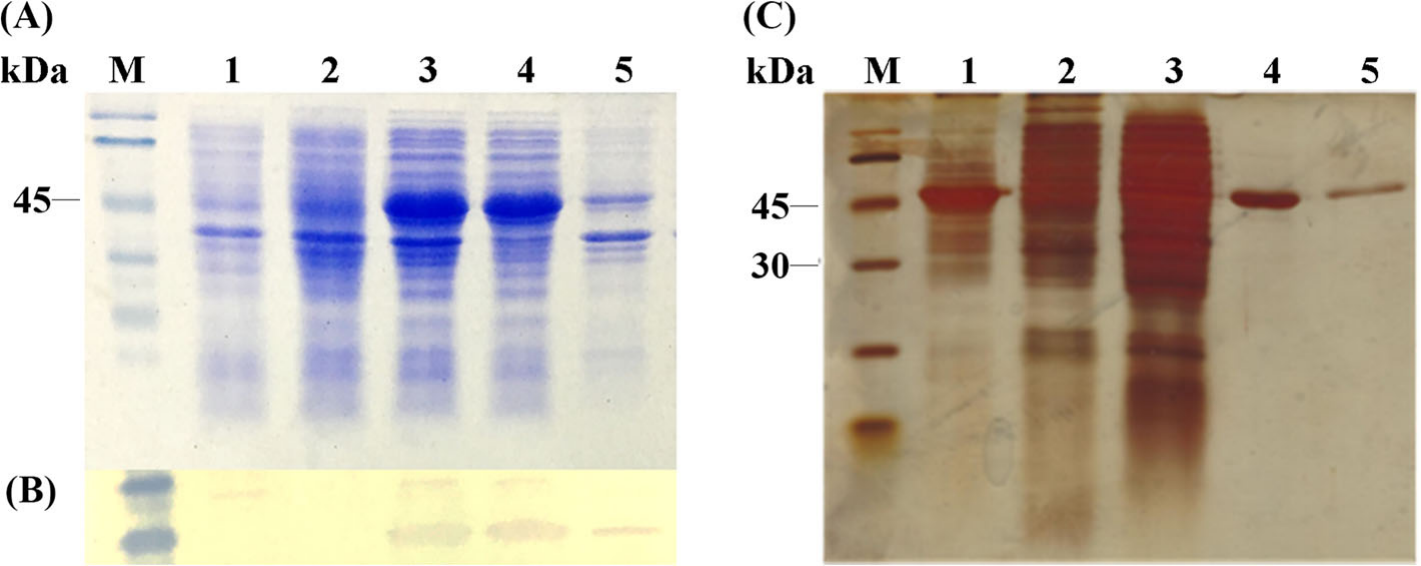
Expression of rPirB^vp^ protein analyzed on SDS-PAGE (**a**), Western blot (**b**), and purification analyzed on SDS-PAGE and silver staining (**c**). **a** and **b** Lane M: protein ladder; Lane 1: *E. coli* BL21(DE3)/pET22b (+IPTG); Lane 2: *E. coli* BL21(DE3)/pET22b (-IPTG); Lanes 3–5: *E. coli* BL21(DE3)/pET22b-PirB^vp^ (+IPTG). **c** Lane M: protein ladder; Lane 1: pre-purified rPirB^vp^ protein; Lanes 2–3: whole cell lysate protein; Lanes 4–5: purified rPirB^vp^ protein

The PirB^vp^ gene was cloned with an His-tag at C-terminal, and the recombinant plasmid construct was then transformed into *E. coli* BL21 (DE3). His-tag was used for the purification of rPirB^vp^ by Ni affinity column. Aliquots of *E. coli*-induced cultures were analyzed on SDS-PAGE (Fig. 2a) and expression of PirB^vp^ protein was confirmed by Western blot probed with anti-His antibody (Fig. 2b). In *E. coli* BL21(DE3)/pET22b-PirB^vp^, PirB^vp^ protein was overexpressed in soluble fraction as a recombinant protein about 50kDa (lane 4, Fig. 2a), a predicted size of PirB^vp^. There was no band of target protein in control samples because they only carried an empty plasmid pET22b, which did not have PirB^vp^ gene. The blotted membrane indicated the presence of corresponding band in the expressed PirB^vp^ protein. The expressed PirB^vp^ protein was purified by HisTrap column and SDS-PAGE analysis with silver staining showed that a relatively high-purity protein was isolated (Fig. 2c).

### Results of immunization and detection of PirB^vp^ protein-specific antibodies

The antisera from immunized rabbits were tested at two-fold serial dilutions for immunoreactivity with recombinant and native PirB^vp^ protein by double immunodiffusion.

**Fig. 3:**
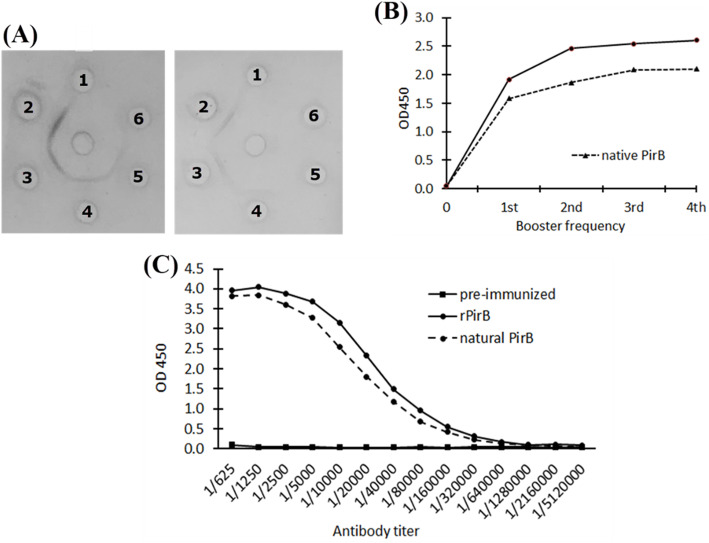
PirB^vp^ protein-specific antibodies evaluation using the Ouchterlony double immunodiffusion technique (**a**), and ELISA for antibody level per booster (**b**), and for antibody titer (**c**). **a** Left image: central well contained rPirB^vp^ protein; Right image: Central well contained native PirB^vp^ protein

This test was used for detection of antibody in rabbit sera which immunized by rPirB^vp^ protein and that has been conducted through the third injection. A control experiment was carried out with pre-immunized rabbit sera. By the naked eye observation, results showed the formation of precipitate lines from sera containing PirB^vp^-specific antibodies but not from pre-immunized rabbit sera (Fig. 3a). The formation of precipitate lines indicated the reaction between given antigen and its specific antibodies formed in immunized rabbit sera.

Evaluation of immune response to the injections was performed by ELISA. Microtiter plates were coated with the recombinant and native PirB^vp^ proteins. The result showed that the level of antibody increased steadily starting from the first injection until the end of the fourth injection as shown in Fig. 3b. Immunized rabbit with rPirB^vp^ protein reacted to native PirB^vp^ protein and the antibody increased after each injection. Antibody peak reached at the third injection and increased slightly after the fourth injection. Therefore, we stopped rabbit immunization at the fourth booster injection. The obtained sera after the second injection was used for the next immunoreactivity.

After the purification of antibody, a serial dilution of antibody from 1:625 to 1:5,120,000 was tested with the recombinant and native PirB^vp^ proteins. Absorbance was measured at 450 nm with a microplate reader, and the titer of each antibody sample was arbitrarily designated as the maximum dilution that yielded at least twice the absorbance of the same dilution of nonimmune control antibody. The result indicated that the mean titer of antibodies was approximately 1:2,560,000 and meanwhile, pre-immunized sera as negative controls did not give a detectable signal (Fig. 3c).

### Characterization of affinity-purified antibodies

**Fig. 4:**
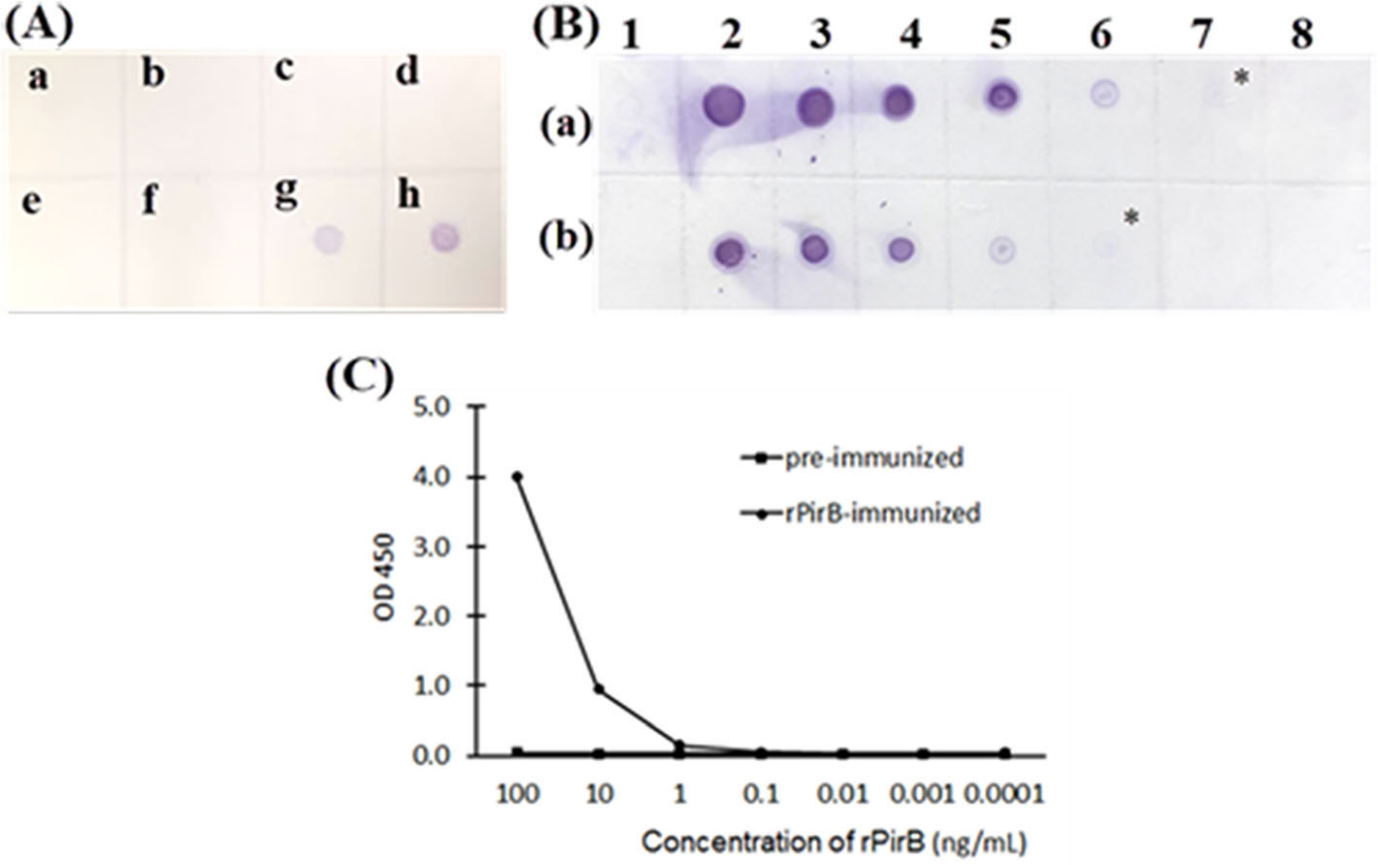
Dot blotting for antibody specificity (**a**), sensitivity (**b**) and ELISA for antibody sensitivity (**c**). (**a**) a: *V. alginolyticus*, b: *V. cholera*, c: *V. vulnificus*, d: VP_non-AHPND_ (XN8), e: White Spot Syndrome virus, f: bovine serum albumin, g: VP_AHPND_ (XN89), h: purified recombinant PirB^vp^ protein. **b** a1: VP_non-AHPND_ (XN8), a2-8: purified rPirB^vp^ protein with 2-fold diluted concentration from 200-3.125 ng/mL, b1: bovine serum albumin, b2-8: native PirB^vp^ of VP_AHPND_ (XN89) with 2-fold diluted concentration from 200 to 3.125 ng/mL

Dot blotting images revealed that the affinity-purified antibodies clearly detected proteins of VP_AHPND_ (XN89), purified recombinant PirB^vp^ protein, and no cross-reactivity with VP_non-AHPND_ (XN8), as well as other *Vibrio* species (*V. alginolyticus, V. cholerae, V. vulnificus*), and White Spot Syndrome virus was observed (Fig. 4a).

Sensitivity of the obtained antibodies was quantified by performing a dot blotting and ELISA. Results from dot blotting showed that the antibodies detected rPirB^vp^ and native PirB^vp^ protein at 6.25 and 12.5 ng/spot, respectively, but not for VP_non-AHPND_ (XN8) (Fig. 4b). Moreover, the results from ELISA showed that the antibodies detected rPirB^vp^ protein at the concentration of 0.1 ng/mL (Fig. 4c).

## Discussion

This study was conducted for production, purification, and evaluation of the rabbit polyclonal antibodies against recombinant PirB^vp^ protein (rPirB^vp^). These antibodies are a powerful tool for developing immunochromatographic test strip of AHPND diagnosis. Initially, after transformation of synthetic gene construct (pET22b/PirB^vp^) to commonly used bacteria *E. coli*, the expression and purification of the recombinant protein were performed and confirmative tests were also carried out. In the previous studies, the rPirB^vp^ protein was expressed with GST-tag [13, 14]. This tag may help maintain the solubility of a fused protein that is normally expressed in an insoluble form [15]. However, the expressed GST fusion protein required several strategic decisions, optimization of methods and conditions for specific proteins including the vector design, expression and purification processes [16]. The GST fusion protein is often expressed at high levels that may result in accumulation of aggregated protein in inclusion bodies [17]. In addition, the large size of the GST-tag and its dimerization in solution may affect the properties of the fusion protein that not easily eluted completely from the bound glutathione agarose [18]. Therefore, GST-tag should be removed after purification when the recombinant proteins used as antigen in producing antibody [19, 20]. In this study, we expressed PirB^vp^ with His-tag because it is one of the most commonly used tag and its small size is weakly immunogenic. So, the fusion protein can be used directly as an antigen for immunization without further purification to remove His tag [18]. To exclude *E. coli* per se or/and sham vector could unexpectedly express protein band(s) that equals to rPirB^vp^, pET22 without carrying PirB^vp^ gene was included in SDS-PAGE analysis. After injecting rPirB^vp^ protein to lab animals, serum antibody titers, isolated from the blood of the animals, were evaluated by ELISA method, and at the end, the purification of polyclonal antibodies against rPirB^vp^ protein was performed by ammonium sulphate precipitation and protein G column. The immunogenic competence of rPirB^vp^ protein was shown in its ability to stimulate humoral response against native PirB^vp^ protein with the titer of antibody reached up to 2,560,000. This titer was higher than published studies [21].

Since the obtained antibodies were pure and it is possible to determine its density, it could also be used in methods of identifying native PirB^vp^ such as ELISA sandwich, and immunochromatographic strip test. With that in mind, western blotting and ELISA were successfully developed for detection of PirA^vp^ and PirB^vp^ based on monoclonal antibodies against the AHPND toxins [9]. However, monoclonal antibody production is significantly more expensive, time- and labor-consuming, and requires additional maintenance of cell lines. Therefore, we used rabbits as host animal for polyclonal antibody production because they have a convenient size, are easy to handle and bleed, and produce adequate volumes of high titer, high affinity, precipitating antisera [21]. Using booster injection also has an effect on the titer of the antibody by maintaining the immune response at an appropriate level [22–24]. Consequently, the limit of detection (LOD) of produced polyclonal antibodies against rPirB^vp^ protein in the ELISA format was 0.1 ng/mL. This LOD value was lower than that of the published study [25]. However, this difference may be attributed to to reagents or/and the instruments used. Polyclonal antibodies demonstrating that they were able to detect native PirB^vp^ protein secreted by VP_AHPND_ bacteria at 12.5 ng by dot blotting. This is a relatively low detection limit compared with the amount of lethal PirB^vp^ protein at 5 μg/g shrimp [26].

## Conclusion

In summary, we have succeeded in expression, purification of the recombinant PirB^vp^ protein, and this protein as an effective immunogen. Polyclonal antibodies were developed from all rabbits immunized with rPirB^vp^ protein which reacted with native PirB^vp^ protein from VP_AHPND_ and no cross-reactivity was observed with other *Vibrio* species and White Spot Syndrome virus as revealed in dot blotting. These antibodies would be useful for further development of lateral-flow strip test.

## Data Availability

All data generated or analyzed during this study are included in this published article.

## Abbreviations

AHPND: Acute hepatopancreatic necrosis disease
ELISA: Enzyme-linked immunosorbent assay
IPTG: Isopropyl-β-D-thiogalactopyranoside
PCR: Polymerase chain reaction
rPirB^vp^: Recombinant PirB^vp^
SDS-PAGE: Sodium dodecyl sulfate polyacrylamide gel electrophoresis
*V.*: *Vibrio*
